# Spillover of Peste des Petits Ruminants Virus from Domestic to Wild Ruminants in the Serengeti Ecosystem, Tanzania

**DOI:** 10.3201/eid2112.150223

**Published:** 2015-12

**Authors:** Mana Mahapatra, K. Sayalel, Murali Muniraju, Ernest Eblate, R. Fyumagwa, S. Shilinde, M. MaulidMdaki, J. Keyyu, Satya Parida, Richard Kock

**Affiliations:** The Pirbright Institute, Woking, UK (M. Mahapatra, M. Muniraju, S. Parida);; Ngorongoro Conservation Area Authority, Arusha, Tanzania (K. Sayalel);; Tanzania Wildlife Research Institute, Arusha (E. Eblate, R. Fyumagwa, S. Shilinde, M. MaulidMdaki, J. Keyyu);; University of London, London, UK (R. Kock)

**Keywords:** peste des petits ruminants virus, viruses, morbillivirus, epidemiosurveillance, wildlife-livestock interface, animals, phylogenetic analysis, ecosystem, Tanzania, Africa, Serengeti, ruminants

## Abstract

We tested wildlife inhabiting areas near domestic livestock, pastures, and water sources in the Ngorongoro district in the Serengeti ecosystem of northern Tanzania and found 63% seropositivity for peste des petits ruminants virus. Sequencing of the viral genome from sick sheep in the area confirmed lineage II virus circulation.

Peste des petits ruminants (PPR) is caused by peste des petits ruminants virus (PPRV), a member of the genus *Morbillivirus* in the family *Paramyxoviridae*, and primarily affects sheep and goats. Although PPRV can infect a wide range of domestic and nondomestic species, the disease has not been confirmed in free-ranging wildlife species in sub-Saharan Africa. Increased understanding of the epidemiology of PPRV infection in mixed species environments is urgently needed, especially because the virus range has apparently expanded in recent years, with associated social and economic effects of epidemics in areas where the disease had not been circulating, including programs for ongoing control (*1*).

PPR was first reported in northern Tanzania in 2008 and resulted from a southward spread from Kenya and Uganda by migrating livestock (*2*,*3*). No serologic and clinical reports of PPRV infection in wildlife occurred in sub-Saharan Africa during 2005–2013, although seropositivity was recorded in Uganda, Ethiopia, and other countries in West and Central Africa before this period (*4*). A study of serum samples collected from 331 wildlife 1–12 years of age in Tanzania, including in the Serengeti, Arusha, Katavi, and Tarangire National Parks and in the Ngorongoro Crater in the Ngorongoro Conservation Area (NCA), showed seronegative results for PPRV (*5*); however, that study did not include animals from our study area. Another study of >500 serum samples from wildlife in northeastern Kenya, tested during 2008–2010 for the Somali Ecosystem Rinderpest Eradication Coordination Unit program, also showed seronegative results for PPRV (F. Gakuya, pers. comm., 2012). In both studies, samples taken in Kenya and Tanzania were banked samples collected opportunistically during other research activities in the study areas. These results indicated that wildlife were not being infected by PPRV in this region; however, because of the opportunistic sampling, more targeted surveillance was considered necessary to confirm this PPRV-seronegative status (*5*). Given the proposed global eradication of PPRV by 2030 (*6*), we sought to determine the role of wildlife as possible hosts and sentinels of PPRV infection in northern Tanzania.

## The Study

**Figure 1:**
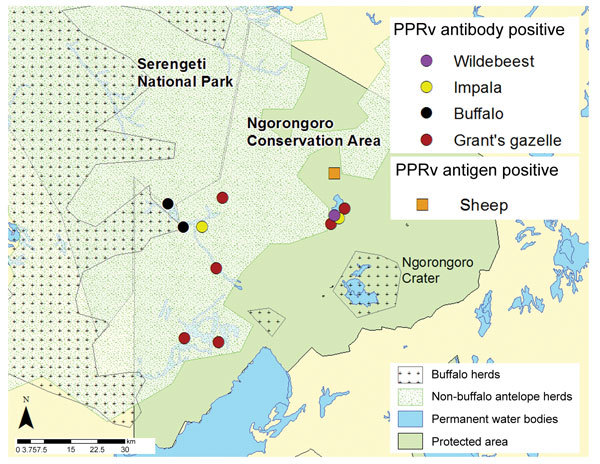
Geospatial map of sampling area showing distribution of buffalo (2014) and nonbuffalo (2006) species in the Greater Serengeti Ecosystem, northern Tanzania. Map is constructed on the basis of aerial census data and sites of livestock and wildlife herds sampled in June 2014 and found to be positive for peste des petits ruminants virus (PPRV) infection in the Ngorongoro Conservation Area.

In June 2014, we investigated whether evidence could be found for PPRV infection in resident wildlife as a result of possible spillover from domestic animals (i.e., nomadic pastoral and agropastoral livestock), with whom they may share pasture and water resources in the Ngorongoro district within NCA (Figure 1). After obtaining ethical approval through the Animal Health and Welfare European Research Area Network; the Biotechnology and Biological Sciences Research Council; and Tanzania government departments responsible for research on wildlife and livestock, we collected serum samples from wildlife near resident livestock or from pastures and water sources shared with resident and nomadic livestock. Sampling was performed during the dry season, so only resident wildlife were sampled; migratory populations had already moved northwest from NCA. Both netting and chemical immobilization were used in 11 sampling sites, and samples were collected from 46 wild animals (Table 1). After the animals were restrained, they were clinically examined, and age was determined on the basis of incisor tooth eruption. Whole blood samples were collected with and without anticoagulant, and eye and nasal swabs were taken.

**Table 1:** Seroprevalence of peste des petits ruminants virus in wildlife in the Ngorongoro Conservation Area, Tanzania, 2014

Species	No. sampled	No. positive/no. negative	Individual prevalence, %	No. herds sampled	No. positive/no. negative	Herd prevalence, %
Buffalo	10	5/5	50	2	2/0	100
Grant’s gazelle	30	20/10	66	8	8/0	100
Thomson’s gazelle	1	0/1	0	1	0/1	0
Wildebeest	2	1/1	50	1	1/0	100
Impala	3	3/0	100	1	1/0	100
Total	46	29/17	63	13	12/13	92

Similar sets of blood, eye, and nasal samples were collected from 5 domestic sheep and 5 goats that were reportedly ill with suspected signs of PPR (2 herds) or opportunistically sampled (1 herd) in locations across the wildlife range, which included 1 resident livestock herd in a high-altitude location and 2 nomadic livestock herds in low-altitude locations. No positive results were obtained from small livestock sampled in the highlands of Ngorongoro Crater. Three sheep (1 young, 1 semi-adult, and 1 adult) from the Esieki plains in the northern part of NCA were fresh cases with PPRV-specific clinical signs; their eye and nasal swab samples tested positive for PPRV antigen by a lateral flow device (*7*) (Table 2; Technical Appendix Figure 1). Although the adult animals had been vaccinated in April 2013, lambs <4 months of age and those born after that month were unvaccinated, creating a window for PPRV infection in the vaccinated herd and indicating a vital need to vaccinate all kids and lambs immediately after weaning, when they lose protection by maternal antibodies. 

**Table 2:** Seroprevalence of peste des petits ruminants virus in domestic small ruminants in the Ngorongoro Conservation Area, Tanzania, 2014

Species	No. sampled	No. positive/no. negative	
		H c-ELISA*	LFD antigen test†
Goat	5	2/0	0/5
Sheep	5	0/2	3/2
Total	10	2/2	3/7

*Serum samples from 2 animals from each species were used to detect antibodies by H c-ELISA (*8*). †Eye and nasal swab samples from 5 animals from each species were used for antigen testing with a lateral flow device (LFD) (*7*).

**Figure 2:**
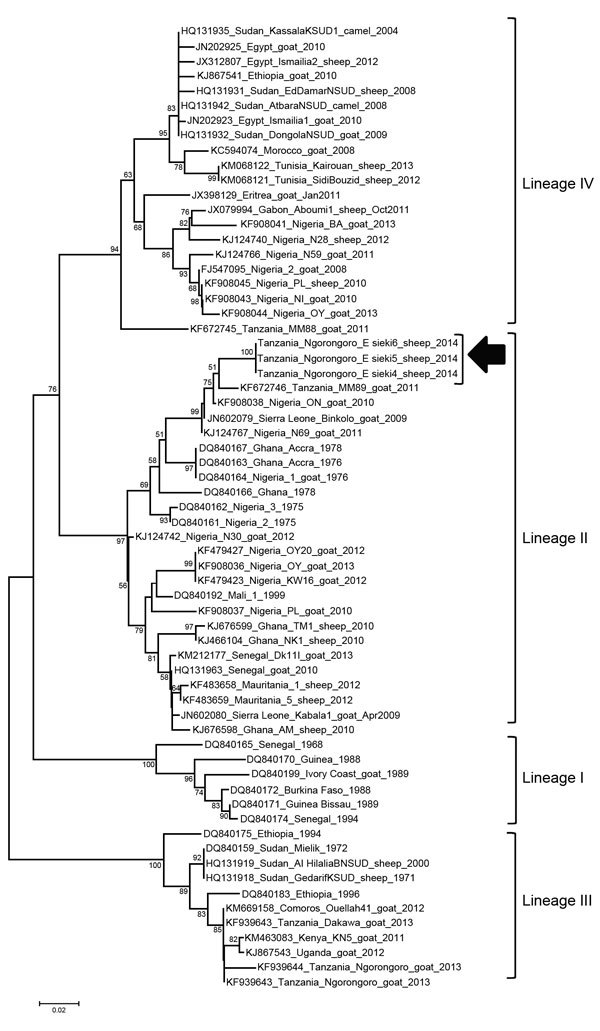
Neighbor-joining tree constructed on the basis of partial N-gene sequences of peste des petits ruminants virus (PPRV), showing relationships among the PPRV isolates from Africa. The Kimura 2-parameter model was used to calculate percentages (indicated by numbers beside branches) of replicate trees in which the associated taxa clustered together in 1,000 bootstrap replicates. Arrow indicates isolates sequenced in this study; sequences have been submitted to GenBank and are awaiting accession numbers. Scale bar indicates nucleotide substitutions per site.

Real-time reverse transcription PCR (*9*) confirmed PPRV infection in all 3 PPRV-positive sheep from the Esieki plains. In addition, a sample from 1 Grant’s gazelle in the Esieki plains was also positive (cycle threshold 34). Samples from 2 domestic young goats from Ngoile, NCA, were also positive (cycle threshold 32 and 37). Amplification of the PPR genome in gel-based PCR was possible only from swabs from the 3 clinically positive animals by using N-gene primers (*10*). The partial N-gene sequences available in GenBank for Africa through December 2014 were aligned and used for constructing a neighborhood-joining phylogenetic tree (Figure 2) that confirmed co-circulation of lineage II PPRV along with lineage III and IV in Tanzania (*11*–*14*). Recently, lineage II has been circulating in Central Africa (*12*,*13*). Possible incursion of lineage II from Central to East Africa, particularly to Tanzania, may have been overlooked because not all outbreaks are reported or investigated by viral genome sequencing.

In addition to infection among domestic livestock, detection of antibodies in blood samples by H c-ELISA (Biological Diagnostic Supplies Ltd., Ayrshire, UK) (*8*) showed PPRV seropositivity in all wildlife species and herds sampled across NCA except for Thomson’s gazelle, but only 1 animal of this species was sampled (Table 1). Age-specific data for the buffalo and Grant’s gazelle showed seropositivity increasing with age (Technical Appendix Figure 2, panels A and B) and included animals <6 months of age.

## Conclusions

Our findings provide evidence for PPRV infection in wildlife in East Africa. Recurring outbreaks in NCA in Tanzania confirm that PPRV, having recently emerged in this region, is likely now endemic in this area and is circulating among sheep, goats, and wildlife despite several rounds of mass vaccination. Most wild ruminant species and sampled subpopulations or herds sharing range with small livestock in NCA have been infected with PPRV, with the youngest wild animal confirmed antibody positive at ≈6 months of age, suggesting recent exposure. 

Our sample represents resident wildlife in NCA and not migrating populations in the ecosystem. The positive result from a small resident herd of wildebeest near Olbalbal warrants closer examination of the PPRV status of migrating populations of wildebeest, Thomson’s gazelle, and topi (a type of antelope, *Damaliscus lunatus*), which moved out of the area during April–May 2014. The single Thomson’s gazelle sample is inconclusive. Age-specific data show that antibody prevalence rises with age, suggesting intermittent but regular exposure in the wildlife populations; however, circulation of the virus within and between the populations of each wildlife species is also possible. 

The transmission and spread of PPRV appears to be considerable; high seroprevalence is observed at individual and herd levels, without all animals being infected, suggesting lower infective loads in the wildlife and a possibility that most infections could result from direct spillover of virus from infected livestock. The possibility of spillover infections is supported by the apparent absence of antibodies in the wildlife populations that have no contact with livestock (*5*). 

Absence of clinical evidence in wildlife does not constitute evidence of absence of the disease. Antibodies were present in many wildlife we sampled, and the genome was present in 1 Grant’s gazelle in the Esieki plains, where ongoing outbreaks were confirmed in domestic sheep. Clinical infections caused by PPRV have been recorded often in captive gazelle (*Gazella* species) (*15*) in United Arab Emirates. Currently, no evidence of wildlife disease exists, but cases or carcasses might go unnoticed because of deaths from other causes and rapid removal of dead animals by scavengers. These findings confirm endemic PPRV in the Greater Serengeti Ecosystem and suggest that free-ranging wildlife are susceptible to infection and can act as sentinels of livestock disease but do not appear to be maintaining infection across their populations. 

**Technical Appendix.** Figures showing additional results of sampling of ruminants for detection of peste des petits ruminants virus infection in Tanzania.
