# Correction: Vol. 21, No. 12

**DOI:** 10.3201/eid2201.C32201

**Published:** 2016-01

**Keywords:** errata, erratum, correction

Several author names were listed incorrectly in Spillover of Peste des Petits Ruminants Virus from Domestic to Wild Ruminants in the Serengeti Ecosystem, Tanzania (M. Mahapatra al.). The article has been corrected online (http://wwwnc.cdc.gov/eid/article/21/12/15-0223_article).

