# Supplementary material for: Spillover of Peste des Petits Ruminants Virus from Domestic to Wild Ruminants in the Serengeti Ecosystem, Tanzania
Source: Emerg Infect Dis. 2015 Dec;21(12):2230–4. doi: 10.3201/eid2112.150223 (PMC4672450; doi:10.3201/eid2112.150223)
Supplement: Supplementary file 1 — Technical Appendix. Figures showing additional results of sampling of ruminants for detection of peste des petits ruminants virus infection in Tanzania. [file 15-0223-Techapp-s1.pdf]

Article DOI: http://dx.doi.org/10.3201/eid2112.150223

Spillover of Peste des Petits Ruminants Virus from Domestic to Wild Ruminants in the Serengeti Ecosystem, Tanzania

Technical Appendix

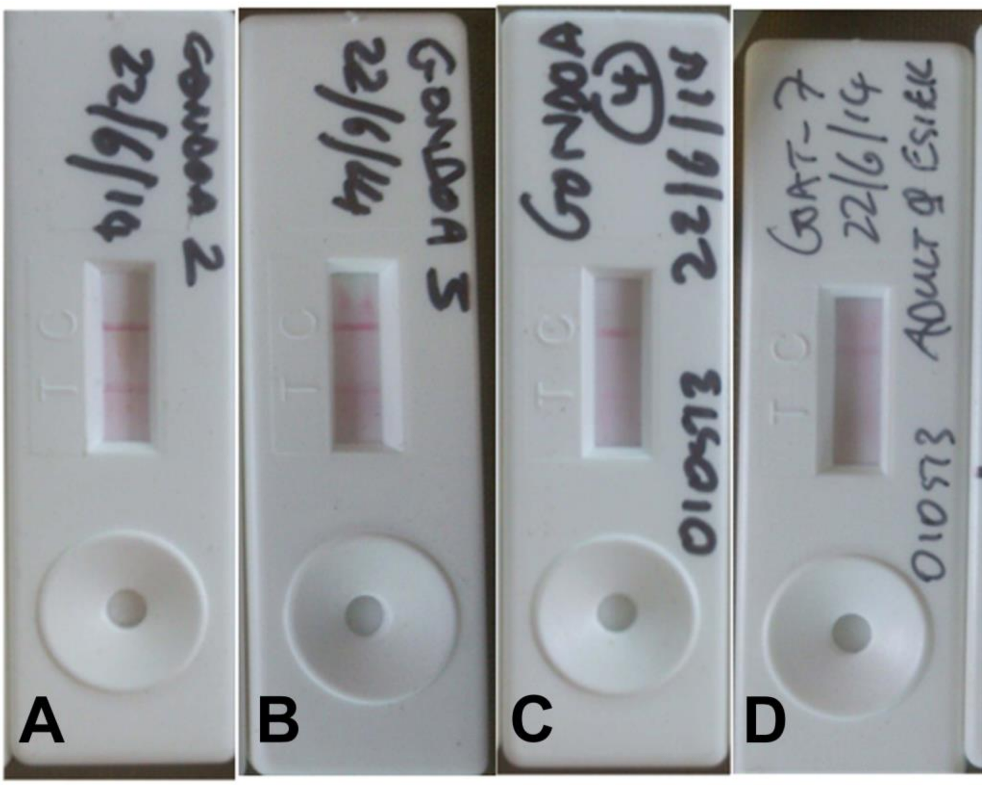

Technical Appendix Figure 1. Screening of swab samples from domestic small ruminants in the Esieki plains for detection of viral antigen by lateral flow device. A-C) Positive samples from sheep as indicated by a dark line under “T” (test); D) negative sample from a goat.

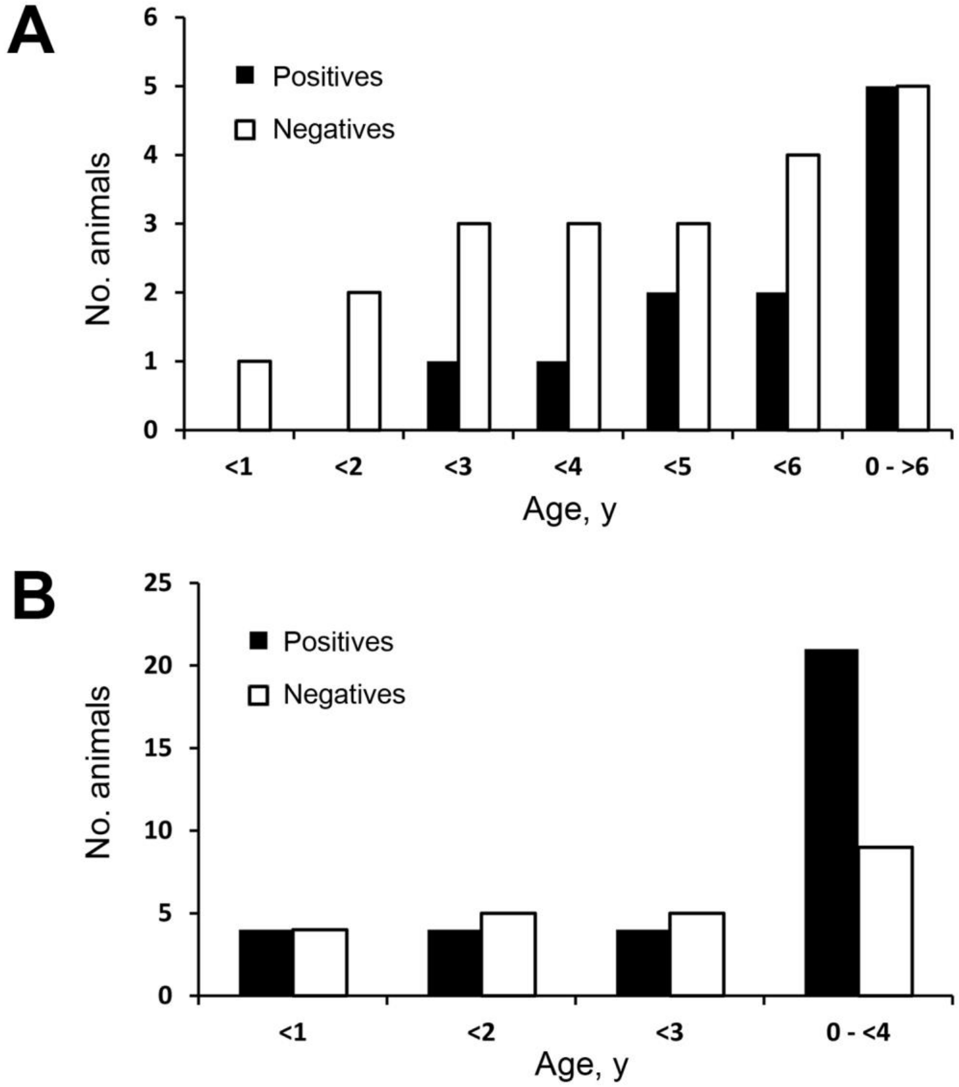

Technical Appendix Figure 2. Results of sampling for selected wildlife in Tanzania, 2014. A) Age-specific, antibody-positive, and negative data for buffalo sampled. B) Age-specific, antibody-positive, and negative data for Grant’s gazelle sampled.
